# Lipopolysaccharide-Induced *CXCL10* mRNA Level and Six Stimulant-mRNA Combinations in Whole Blood: Novel Biomarkers for Bortezomib Responses Obtained from a Prospective Multicenter Trial for Patients with Multiple Myeloma

**DOI:** 10.1371/journal.pone.0128662

**Published:** 2015-06-26

**Authors:** Takashi Watanabe, Masato Mitsuhashi, Morihiko Sagawa, Masaki Ri, Kenshi Suzuki, Masahiro Abe, Ken Ohmachi, Yasunori Nakagawa, Shingen Nakamura, Mizuki Chosa, Shinsuke Iida, Masahiro Kizaki

**Affiliations:** 1 Hematology Division, National Cancer Center Hospital, Tokyo, Japan; 2 Hitachi Chemical Research Center, Inc., Irvine, California, United States of America; 3 Department of Hematology, Saitama Medical Center, Saitama Medical University, Kawagoe, Japan; 4 Department of Medical Oncology and Immunology, Nagoya City University Graduate School of Medical Sciences, Nagoya, Japan; 5 Department of Hematology, Japanese Red Cross Medical Center, Tokyo, Japan; 6 Department of Hematology, Endocrinology and Metabolism Instutute of Biomedical Sciences, Tokushima University Graduate School, Tokushima, Japan; 7 Division of Hematology/Oncology, Department of Internal Medicine, Tokai University School of Medicine, Isehara, Japan; IIT Research Institute, UNITED STATES

## Abstract

To identify predictive biomarkers for clinical responses to bortezomib treatment, 0.06 mL of each whole blood without any cell separation procedures was stimulated *ex vivo* using five agents, and eight mRNAs were quantified. In six centers, heparinized peripheral blood was prospectively obtained from 80 previously treated or untreated, symptomatic multiple myeloma (MM) patients with measurable levels of M-proteins. The blood sample was procured prior to treatment as well as 2-3 days and 1-3 weeks after the first dose of bortezomib, which was intravenously administered biweekly or weekly, during the first cycle. Six stimulant-mRNA combinations; that is, lipopolysaccharide (LPS)-*granulocyte-macrophage colony-stimulating factor (GM-CSF)*, LPS-*CXCL chemokine 10 (CXCL10)*, LPS-*CCL chemokine 4 (CCL4)*, phytohemagglutinin-*CCL4*, zymosan A (ZA)-*GMCSF* and ZA-*CCL4* showed significantly higher induction in the complete and very good partial response group than in the stable and progressive disease group, as determined by both parametric (*t*-test) and non-parametric (unpaired Mann-Whitney test) tests. Moreover, LPS-induced *CXCL10* mRNA expression was significantly suppressed 2-3 days after the first dose of bortezomib in all patients, as determined by both parametric (*t*-test) and non-parametric (paired Wilcoxon test) tests, whereas the complete and very good partial response group showed sustained suppression 1-3 weeks after the first dose. Thus, pretreatment LPS-*CXCL10* mRNA and/or the six combinations may serve as potential biomarkers for the response to bortezomib treatment in MM patients.

## Introduction

The proteasome inhibitor bortezomib (VELCADE; Millennium Pharmaceuticals and Johnson & Johnson Pharmaceutical Research & Development) has revolutionized the treatment of multiple myeloma (MM) patients and has become a mainstay in the standard of care for both previously untreated [1] and relapsed [2, 3] patients with MM. A number of clinical and laboratory features provide prognostic information for patients with MM, such as hypodipoidy [4] and chromosomal translocations and deletions [5–7].

The gene expression profiles of plasma cells isolated from the bone marrow of MM patients can predict the response to treatment with bortezomib [8, 9]. However, peripheral blood (PB) biomarkers able to predict the response to bortezomib have not yet been identified, although some factors are known to correlate with such responses, including hepatocyte growth factor [10], thrombospondin [10], XBP-1 [11] and absolute lymphocyte counts [12].

In our previous study [13], we reported that phytohemagglutinin (PHA)-induced *interleukin-2 (IL2)* mRNA levels in *ex vivo* whole blood obtained prior to bortezomib treatment could predict the incidence of bortezomib-induced peripheral neuropathy. In this study, we used the same assay to predict the efficacy of bortezomib treatment in an expanded patient population.

## Subjects and Methods

### Patients

Eligible patients in this multicenter prospective study consisted of previously treated MM patients or untreated patients with symptomatic MM, as described in our previous study [13]. All patients had to have measurable levels of M-proteins. The study was approved by the institutional review board or independent ethics committee at all participating institutions and was conducted according to the principles of the Declaration of Helsinki and the International Conference on Harmonization Guidelines of Good Clinical Practice. All patients provided written informed consent for sample procurement. The following institutions participated in this study: National Cancer Center Hospital; Saitama Medical Center, Saitama Medical University; Nagoya City University, Graduate School of Medical Sciences; Japanese Red Cross Medical Center; University of Tokushima, Graduate School of Medical Sciences and Tokai University School of Medicine (Acknowledgement section of the ms.). Clinical responses were assessed according to the International Uniform Response Criteria [14].

### Measurements

Eight-well strips containing 1.2 μL each of PHA (2 mg/mL), heat-aggregated immunoglobulin G (HAG) (10 mg/mL), lipopolysaccharide (LPS) (0.5 mg/mL), zymosan A (ZA) (75 mg/mL) or solvent phosphate-buffered saline (PBS) were delivered to each institution on dry ice. These strips were kept frozen at -80°C. A 2 mL sample of heparinized PB was obtained from each patient prior to treatment as well as 2–3 days (D2-3) and 1–3 weeks (W1-3) after intravenous administration of the first dose of bortezomib during the first cycle. The blood was immediately delivered to the designated laboratory, 0.06 mL of PB was added to each well containing 3 strips (that is, in triplicate), and the strips were incubated for 4 hours at 37°C. The total blood volume required was 0.9 mL (0.06 mL/well x 5 wells/strip x 3 strips). After incubation, the samples were stored at -80°C.

### mRNA analysis

Purification of mRNA and cDNA synthesis were performed as described previously using leukocyte capture filter plates and oligo(dT)-immobilized microplates [15,16] The cDNA was used for real-time PCR [15,16]. Melting curves were analyzed to confirm that the PCR signals were derived from a single PCR product, and the cycle threshold (Ct) value was determined using analytical software (SDS, Thermo Fisher Scientific, Carlsbad, CA). The Ct values of the treated samples were subtracted individually from the mean Ct values of the control samples to calculate the ΔCt, and the fold increase was calculated as 2^(-ΔCt), as described previously [15,16]. The mRNAs analyzed included *β-actin* (*ACTB*), *IL2* and *interleukin-6* (*IL6*), *granulocyte-macrophage colony-stimulating factor* (*GMCSF*), *interferon-γ* (*IFNG*), *tumour necrosis factor-α* (*TNFSF2*), *CCL chemokine 4* (*CCL4*) and *CXCL chemokine 10* (*CXCL10*) [16]. mRNA analysis and clinical data collection were performed separately at the different centers.

### Statistical analyses

Parametric (*t*-test) and non-parametric (unpaired Mann-Whitney and paired Wilcoxon tests) tests were used to compare mRNA levels between the two groups. p<0.05 were considered significant. The statistical analyses were performed using Excel (Microsoft, Redmond, WA) and Prism 6 (GraphPad Software, La Jolla, CA).

## Results

### Patients’ charasteristics

Between March 2010 and March 2012, a total of 83 patients (44 male and 39 female) were enrolled from six centers. The median age of all patients was 63 years (Table 1). Fifty patients were previously treated, and 33 patients were untreated. After excluding one patient who died early from progressive disease, another who received additional treatment and another who committed suicide, 80 patients were eligible for response analysis. The numbers of patients who demonstrated complete response (CR), very good partial response (VGPR), partial response (PR), stable disease (SD) and progressive disease (PD) were 5, 7, 33, 33 and 2, respectively.

**Table 1:** Patients Demographic and Baseline Characteristics.

Characteristics		Number of patients(%)
Age, years		
	Median	63
	Range	37–79
Male sex		44(53)
Prior therapy		
	Yes	50(60)
	No	33(40)
M component		
	IgG	48(58)
	IgA	9(11)
	IgD	3(4)
	Light chain only	23(28)
ISS stage		
	I	25(30)
	II	29(35)
	III	29(35)
Follow-up*, days		
	Median	151
	Range	26–666
Bortezomib administration		
	Twice-weekly	63(76)
	Weekly	20(24)
Concurrent dexamethasone		
	Yes	74(89)
	No	9(11)
Best response to treatment†		
	CR	5(6)
	VGPR	7(8)
	PR	33(40)
	SD	33(40)
	PD	2(2)
	NE	3(4)‡

CR, complete response; ISS, International Staging System; NE, not evaluable; PD, progressive disease; SD, stable disease; VGPR, very good partial response.

*Excluded were three patients not evaluable for response.

^†^According to the International Uniform Response Criteria (Durie et al, 2006).

^‡^One patient died of progressive disease early, another received additional chemotherapy, and the third committed suicide.

### mRNA analysis

Overall, 3,600 mRNA preparations and cDNA synthesis reactions were carried out (80 patients x 5 stimulants x 3 time points x 3 [triplicate]). A total of 28,800 PCR reactions were performed (3,600 cDNA samples x 8 genes).

### Pretreatment higher induction of LPS/ZA-induced *GMCSF*, LPS-induced *CXCL10*, and LPS/PHA/ZA-induced *CCL4* mRNA in CR/VGPR responders to bortezomib

**Fig 1:**
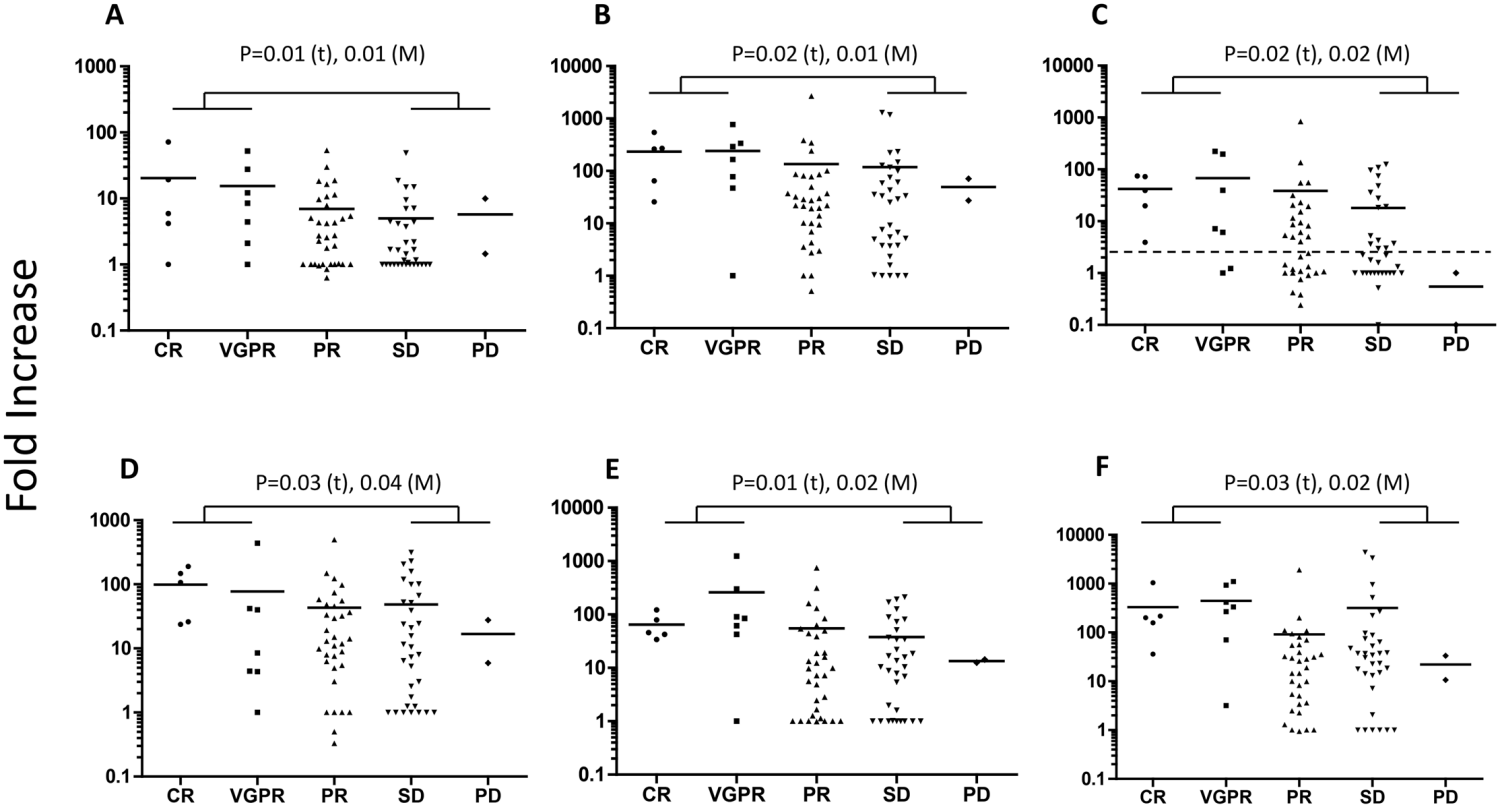
*Ex vivo* mRNA induction in blood obtained prior to bortezomib treatment. The fold increase in (A) LPS-induced *GMCSF*, (B) ZA-induced *GMCSF*, (C) LPS-induced *CXCL10* (top panel), (D) PHA-induced *CCL4*, (E) LPS-induced *CCL4* and (F) ZA-induced *CCL4* (lower panel) mRNA in the CR, VGPR, PR, SD and PD groups is shown. The statistically significant difference between the CR+VGPR and SD+PD groups is shown. t: Student’s *t*-test, M: Mann-Whitney test. Dotted line: fold increase = 3. Samples showing a fold increase in *ACTB* (which was > 3) were removed from the analysis. Horizontal bars: the mean values.

The fold increase in LPS-induced *GMCSF*, *CXCL10* and *CCL4*, PHA-induced *CCL4* and ZA-induced *GMCSF* and *CCL4* were significantly higher in the CR and VGPR groups than in the SD and PD groups, as determined by both parametric *t*-tests and non-parametric Mann-Whitney tests, whereas the PR group exhibited an intermediate value (Fig 1). Moreover, 100, 67, 56, 42 and 0% of patients showed more than 3-fold increases in LPS-induced *CXCL10* (dotted line in Fig 1).

### Sustained suppression of LPS-induced *CCCL10* mRNA in CR/VGPR responders to bortezomib

**Fig 2:**
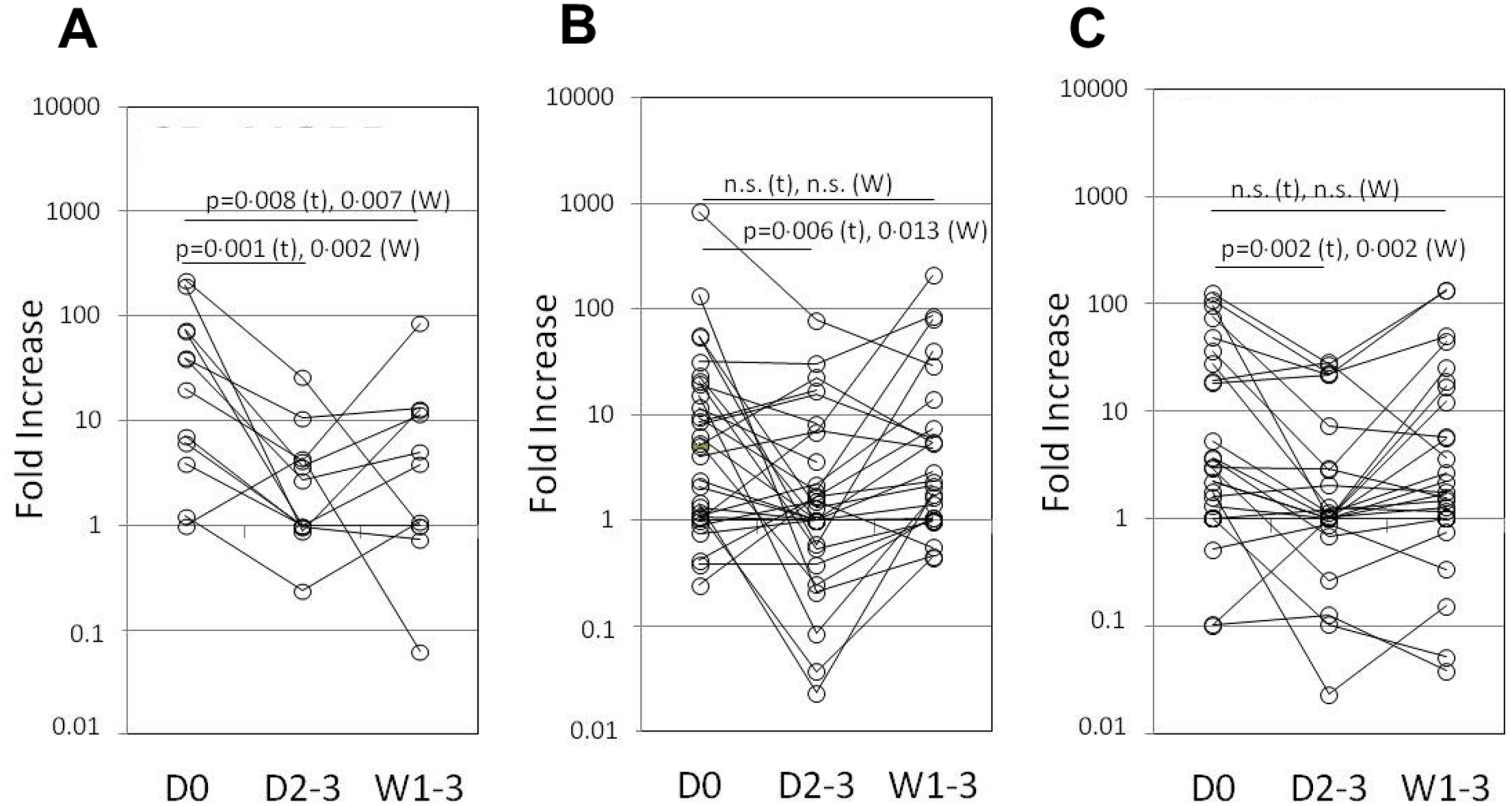
LPS-induced *CXCL10* expression before and after bortezomib treatment. Each point/line represents the fold increase in LPS-induced *CXCL10* expression in each patient in the (A) CR+VGPR, (B) PR and (C) SD+PD groups. The statistically significant difference between the pretreatment (D0) and 2–3 days (D2-3) or 1–3 weeks (W1-3) after intravenous administration of the first dose of bortezomib during the first cycle groups is shown. t: Student’s *t*-test, W: Wilcoxon test.

As shown in Fig 2, LPS-induced *CXCL10* mRNA expression was significantly suppressed 2–3 days after the first dose of bortezomib in all groups, as determined by both parametric (*t*-test) and non-parametric (paired Wilcoxon test) tests, while the CR+VGPR group showed sustained suppression even 1–3 weeks after treatment. This significant level of inhibition was only observed for LPS-induced *CXCL10*.

## Discussion

Currently, triple drug combinations are believed to be very effective for MM treatment based on the results of several randomised clinical trials [1,17–23] and phase I/II studies [24, 25]. However, in some studies, the daily dose of bortezomib [1, 26–28] and the bortezomib administration schedule (namely, the dose density) [29–31] are reduced to avoid toxicity, despite the fact that bortezomib accumulation is important for achieving improved survival [32]. Although MM patients still experience relapse or progression even after triple drug combination therapy, it is clear that better responses to initial therapy result in longer survival [33–36]. If we could predict which patients will respond to bortezomib, we would be able to give priority to bortezomib dose, rather than the other drugs, in combination regimens. Thus, it is important to predict whether patients will respond to bortezomib before initiation and whether bortezomib can be used in consolidation [1, 37] or maintenance [29, 30, 38, 39] therapy.

This is the first report demonstrating the use of LPS-induced *CXCL10* mRNA levels as a biomarker for assessing the clinical response to bortezomib treatment in PB. We showed that higher induction of *CXCL10* mRNA corresponded to very good responses (CR+VGPR), whereas lower induction corresponded to poor responses (SD+PD); the values in the PR group were variable. Moreover, LPS-induced *CXCL10* mRNA was significantly and continuously suppressed in the good response group even 1–3 weeks after treatment, whereas in the other groups, the suppressive activity was transient and *CXCL10* mRNA levels returned to the original values. It could be argued that the best biomarker may be ZA-induced *GMCSF* because the mean mRNA levels appeared to be higher in both the CR and VGPR groups than those in other response groups. The range of LPS-induced *CXCL10* expression in the CR group was within the ranges of LPS-induced *CXCL10* expression in both PR and SD groups, and lack of overlap was seen in only the patients with CR versus the two patients with PD, although the latter was a small number. Similarly, LPS-induced *CXCL10* expression in the VGPR group generally fell within the ranges seen in the patients with PR and those with SD. However, the majority of MM patients will respond to bortezomib especially in case used as the first-line treatment. Therefore, it is more important to distinguish the patients with PD after treatment with bortezomib, which may have the mutations as to the proteasome pathway [40], from responders to bortezomib rather than finding the difference in the response.

CXCL10, which was previously referred to as interferon γ-inducible 10 kDa protein (IP-10), belongs to the C-X-C family of chemokines that cluster on human chromosome 4 (q12-21). CXCL10 acts as a chemoattractant for human monocytes, activates T cells through binding to the CXCR3 receptor and promotes T cell adhesion to endothelial cells [41]. CXCL10 also elicits a Th1 cell-dominated anti-tumour inflammatory response that can inhibit plasmacytoma growth [42]. Moreover, activated tumour-specific T cells that express CXCR3 were shown to infiltrate CXCL10-expressing myeloma cells more efficiently than non-CXCL10-expressing myeloma cells [43]. CCL4 is another chemotactic factor, and GMCSF is a growth factor for antigen-presenting cells. Thus, the higher induction of *CXCL10*, *CCL4* and *GMCSF* mRNA exhibited by the good responder group (Fig 1) was not unexpected and suggests that these patients may have greater anti-tumour immunity.

However, after bortezomib was administered to MM patients, *CXCL10* mRNA induction was significantly suppressed, and sustained suppression correlated with good responses to treatment (Fig 2). Usually bortezomib may be administerd to MM patients in combination with dexamethasone (the same day as that of bortezomib administration and the subsequent day) as used in the SUMMIT trial [44]. Actually the majority of the patients enrolled in this study received dexamethasone (Table 1) in the above-mentioned way, although detailed data were collected but not shown. In case of dexamethasone-naïve patients with MM, they can still respond to dexamethasone. Consequently, we may not be able to distinguish the responders to bortezomib from the responders to dexamethasone in 2–3 days after the first dose of bortezomib. Therefore, the induced mRNA that caused demonstrable sustained suppression may indicate more meaningful predictor of bortezomib responders. In addition, as mentioned above, such triple combination as bortezomib, melphalan, and prednisone is believed to be best among double to quadruple combinations and therefore used commonly, and melphalan and prednisone will be given days 1–4 of each cycle [1, 17]. In those cases, afore-mentioned, sustained suppression might become powerful tool of prediction although further studies in this approach in combination trials is essential as a validation exercise for theses assays to go forward.

**Fig 3:**
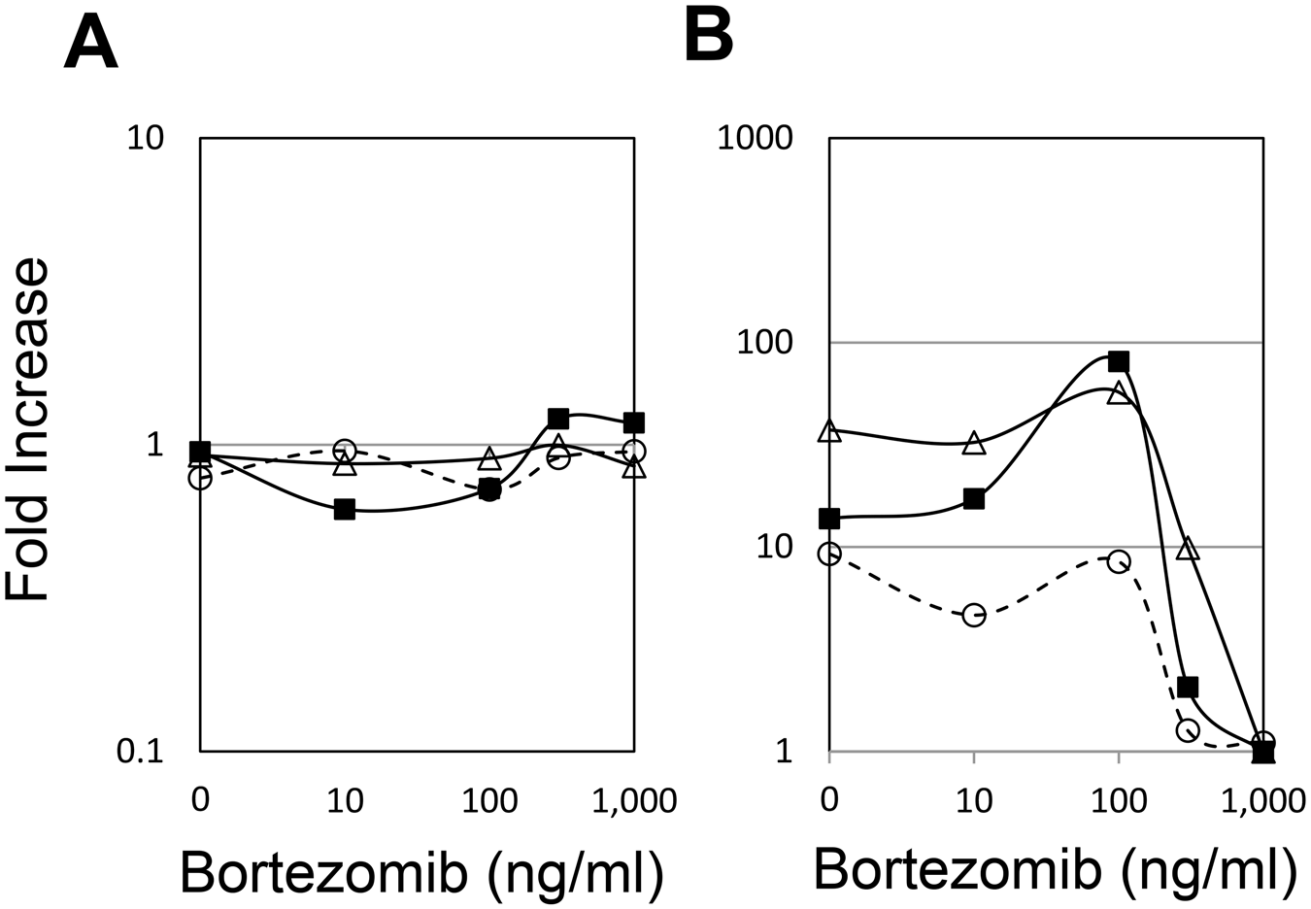
Bortezomib-induced inhibition of LPS-induced *CXCL10* mRNA *ex vivo*. Peripheral blood obtained from 3 healthy volunteers was pre-treated with various concentrations of bortezomib for 1 h and then further stimulated with LPS or PBS (as the control) for an additional 4 h. The fold increase in (A) *ACTB* and (B) *CXCL10* expression is shown. Each symbol represents a single individual.

This observation was likely not explained by the immunological activity of CXCL10, as CXCL10 is also expressed in human myeloma cell lines [45] and is known to stimulate myeloma cell migration [46] and adhesion to bone marrow stromal cells [47]. Thus, MM cells may be more susceptible to the bortezomib-induced inhibition of *CXCL10* mRNA expression than immune cells. Moreover, when bortezomib was added to whole blood prior to LPS stimulation *ex vivo*, *CXCL10* mRNA induction was inhibited in a dose-dependent manner (Fig 3). Thus, LPS-induced *CXCL10* expression in *ex vivo* blood samples could serve as a surrogate marker for the effect of bortezomib *in vivo*.
